# Unconfined Compressive Strength of Cement-Stabilized Qiantang River Silty Clay

**DOI:** 10.3390/ma17051082

**Published:** 2024-02-27

**Authors:** Lisha Zhang, Yuan Li, Xiao Wei, Xin Liang, Jinhong Zhang, Xuchen Li

**Affiliations:** 1 Department of Civil Engineering, Hangzhou City University, Hangzhou 310015, China; zhangls@hzcu.edu.cn (L.Z.);; 2 Key Laboratory of Safe Construction and Intelligent Maintenance for Urban Shield Tunnels of Zhejiang Province, Hangzhou 310015, China; 3 College of Civil Engineering and Architecture, Zhejiang University, Hangzhou 310058, China; 4 Zhejiang Province Institute of Architectural Design and Research, Hangzhou 310006, China

**Keywords:** cemented soil, sustainable dosage design, curing time, unconfined compressive strength

## Abstract

Cement-stabilization of weak and soft soils is an efficient way for ground improvement. Traditional Portland cement remains the most popular cementitious material in practice, and thus, a proper dosage design of cement-stabilized soil is of practical interest to meet the sustainable engineering requirements and to remedy environmental concerns. Based on the unconfined compression test of cement-stabilized Qiantang River silty clay, the effects of cement content, mixing moisture content, mixing-water-to-cement ratio, and curing time on the unconfined compressive strength were investigated. The results show that the mixing-water-to-cement ratio can comprehensively characterize the effects of cement content and water content on the unconfined compressive strength of the cement-stabilized clay. A prediction method for the unconfined compressive strength of cement-stabilized Qiantang River silty clay has been proposed with considerations for mixing-water-to-cement ratio and curing time. By comparing the experimental data of the present study with the existing literature data, it is found that there is a unified relationship between the unconfined compressive strength and the mixing-water-to-cement ratio of cement-stabilized Qiantang River silty clay, kaolin, Singapore marine clay, and Bangkok clay under the same curing time. The prediction method recommended by the standard may overestimate the unconfined compressive strength of cement-stabilized Qiantang River silty clay cured for 90 days.

## 1. Introduction

Soft clays, commonly with low shearing strength and high volumetric compressibility, are widely distributed in the alluvial plains in the east of China, which need to be improved to ensure their engineering properties meet the design requirement before carrying out any construction projects. Among various types of ground improvement methods, cement stabilization can effectively increase shearing strength and compressive stiffness within a relatively short period [1,2]. However, the most popular cementitious material for ground improvement remains the traditional Portland-type cement with a high carbon footprint, though researchers have extensively investigated many types of low-carbon cement. To meet both the engineering demands and the environmental requirements, a proper dosage design of the cement-stabilized soil is of practical interest to avoid excessive cement usage.

The unconfined compressive strength (UCS) of cement-stabilized soil is a major index in engineering design, since it is directly related to the bearing capacity of the stabilized foundation and many other engineering properties [3]. However, USC can be affected by a variety of factors, including the type of soil, cement content, water content, water-to-cement ratio, curing time, types of admixtures, etc. [4,5,6,7,8,9]. There is no universal method to predict the UCS of cement-stabilized soils. 

It is widely accepted that UCS increases with increasing cement content and decreases with increasing mixing-water content under otherwise similar conditions. Tan et al. [10] improved Singapore marine clay using cement mixing and found that the UCS of the specimens prepared with the same water content increases with increasing cement content in a linear pattern. Horpibulsuk et al. [5] reported experimental data showing that the UCS of cement-stabilized Bangkok clay increases with increasing cement content for the same water content of 80% and all the tested curing durations, while they also reported a set of experimental data of cement-stabilized Ariake clay showing that the UCS decreases with increasing water content for a given cement content for all tested curing durations. 

The combined impacts of cement content and water content on UCS can be characterized using the water-to-cement ratio (*w*/*c*) of the mixture of water, cement, and clay in a more or less unified way [5,11]. This resembles Abram’s law, which is widely observed in the concrete industry. Horpibulsuk et al. [5] proposed that the UCS of cement-stabilized clay at high water contents decreases with increasing *w*/*c* in an exponential function, regardless of the cement content and water content. Several subsequent investigations [12,13] further confirmed that *w*/*c* is the only governing parameter on the UCS of cement-stabilized clays prepared at a water content higher than the optimum water content. However, Lee et al. [6] found that the UCS of cement-stabilized Singapore marine clays decreases with increasing *w*/*c* ratio, but the mass ratio between the cement and the soil also has a certain level of impact on the UCS when compared at the same *w*/*c*. Similar observations were also reported by other researchers [10,14,15]. 

In addition to the characterizing methods adopting mixing ratios, such as cement content and water-to-cement ratio, several other indices have been proposed with considerations of void space and bonding properties. Lorenzo and Bergado [7,16] found that after curing the void ratio and cement content can characterize the strength of the cemented clay. Consoli et al. [17,18] suggested that the ratio between the volume of voids and the volume of cement, which is equivalent to *n*/*C*_iv_ (*n* is the porosity and *C*_iv_ is the volumetric content of cement), is the dominant factor controlling the strength of cemented soils for a given mixing-water content, and correlations between UCS and *n*/*C*_iv_ (or its alternatives, *n*/(*C*_iv_)*^m^*, where *m* is a fitting constant) have been proposed for a variety of cement-stabilized soils [19] and soils stabilized using other types of binder materials [20]. Jongpradist et al. [21] proposed the concept of effective void ratio to evaluate a series of mechanical properties of cemented soils including UCS, and it can characterize the effects of cement content, mixing-water content, void ratio, and curing duration on UCS and other mechanical properties. 

According to the above literature, the dosage-based and the void-based characterizations of the UCS are more or less soil-specific. This is probably due to different mineralogies and constituents of the soils. Bell [1] indicated that soils with higher clay content generally require more cement to achieve the desired strength after improvement. Bergado et al. [2] suggested that a higher clay content and plasticity index can have a negative impact on the cement stabilization of soil. Chian et al. [15] systematically investigated the sand content on the UCS of cemented Singapore marine clay and found that UCS decreases with increasing *w*/*c*, but UCS remains the function of the soil-to-cement ratio for a given *w*/*c*. In addition, the impact of the soil-to-cement ratio on UCS can be either positive or negative depending on the sand content. Noting that the mineralogy and constituents of soils can vary spatially at a given site and differ among different sites, empirical values and predictions obtained from cemented soils at other sites may not be applied to the current projects. 

This study conducted a series of unconfined compression tests to evaluate the UCS of cemented Qiantang River silty clay prepared with different cement contents and water contents. A prediction method for the unconfined compressive strength of cement-stabilized Qiantang River silty clay has been proposed with considerations of mixing-water-to-cement ratio and curing time. In addition, the experimental data of this study are compared with the literature data to propose a common zone for characterizing the UCS of cemented clay using the water-to-cement ratio. Discussions are also presented to suggest possible modifications for a practical standard. 

## 2. Experimental Program

### 2.1. Testing Materials

**Figure 1:**
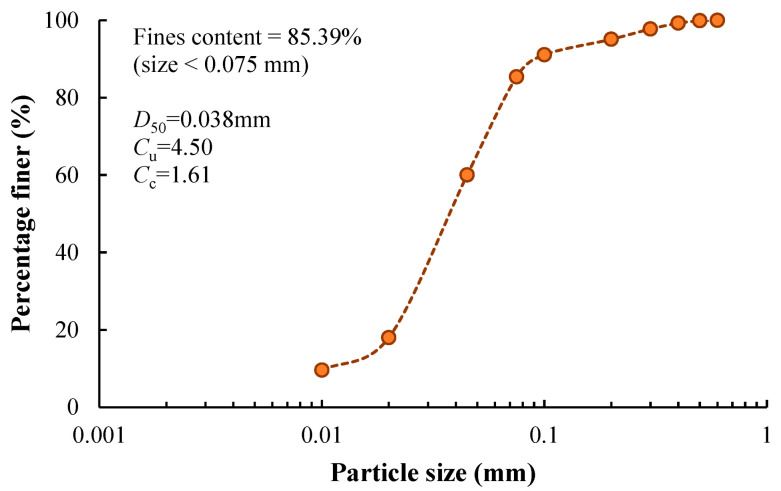
Particle size distribution of Qiantang River silty clay.

**Figure 2:**
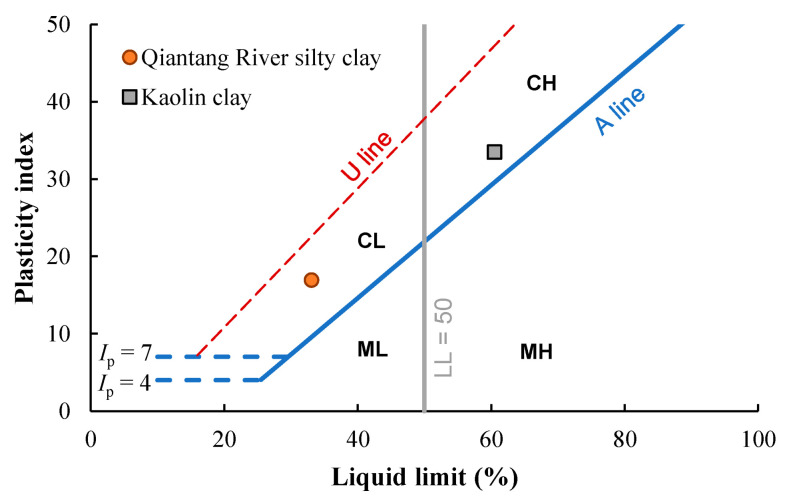
Plasticity chart of the Qiantang River silty clay and commercial Kaolin clay.

The soil used in this study was obtained at an excavation near the Qiantang River, Hangzhou. Its particle size distribution curve (Figure 1) was determined using a laser particle size analyzer, using a mixture of soil and water with sodium hexametaphosphate as the dispersion agent. The result shows that the soil contains 85.4% fines (particles with size < 0.075 mm), and the mean particle size is 0.038 mm. The liquid limit (*w*_L_) and the plastic limit (*w*_P_) are 33.1% and 16.2%, respectively. The physical properties of the silty clay are summarized in Table 1, together with a commercial kaolin clay used by the authors for comparison. By plotting the plastic index (*I*_p_) against *w*_L_ in the plasticity chart (Figure 2), the soil is lean clay (CL). In comparison, Figure 2 also includes the data from the Kaolin clay. The cement is ordinary Portland cement (known as PO42.5R produced by Hailuo Company, Anhui, China), with around 80% of cement clinker, 5% of gypsum, and 14% of mixtures of calcite, fly ash, and slag. A recent study using the same type of cement provided the chemical composition of the cement [22]. It should also be noted that the chemical composition could affect the UCS of cement-stabilized clay and its long-term development [22,23].

### 2.2. Testing Procedures

A certain amount of oven-dried soil was mixed with water and cement using a mechanical mixer to form a uniform mixture within 15 min. The amounts of water and cement were calculated based on the testing scheme, which will be introduced later. This type of sample-preparation method is commonly used in the literature for cement-stabilized clays [24,25], and it is considered suitable for the dosage design of projects involving techniques such as deep cement mixing and jet grouting. The mixture was poured into the mold with an inner diameter of 50 mm and a height of 100 mm, to fill the mold with two layers of slurry. The filled mold was vibrated for 2 min to eliminate air bubbles on a vibration table. Then, the surface of the specimen was leveled and the mold was sealed for curing. The specimens were cured underwater at a temperature of 25 °C. When cured to the prescribed time, the specimens were retrieved from the mold. After measuring the diameter, height, and weight of the specimens, they were compressed to failure using a universal loading machine under an unconfined condition at a loading rate of 1 mm/min. For each mixing ratio, at least three specimens were tested. If the UCS of the three specimens deviated from the average value by ±10%, additional specimens were tested. 

### 2.3. Testing Scheme

This study considers three important factors in engineering design, namely cement content, water content, and curing duration. Three cement contents (*cc*), two mixing-water contents (*w*), and four curing durations (*T*) were adopted, namely, *cc* = 20, 30, and 40%, *w* = 35% and 42%, *T* = 3, 7, 14, and 28 days. The cement content is the ratio between the dry mass of added cement to the dry mass of the soil. The mixing-water content is the ratio of the total mass of water in the slurry to the dry mass of the soil during mixing. The mixing-water-to-cement ratio (*w*/*c*), which is the ratio between the total mass of water in the slurry to the dry mass of cement, can be calculated as *w*/*c* = *w*/*cc*. Table 2 summarizes the testing scheme. 

## 3. Test Results

### 3.1. Effects of the Mixing Ratio on UCS

**Figure 3:**
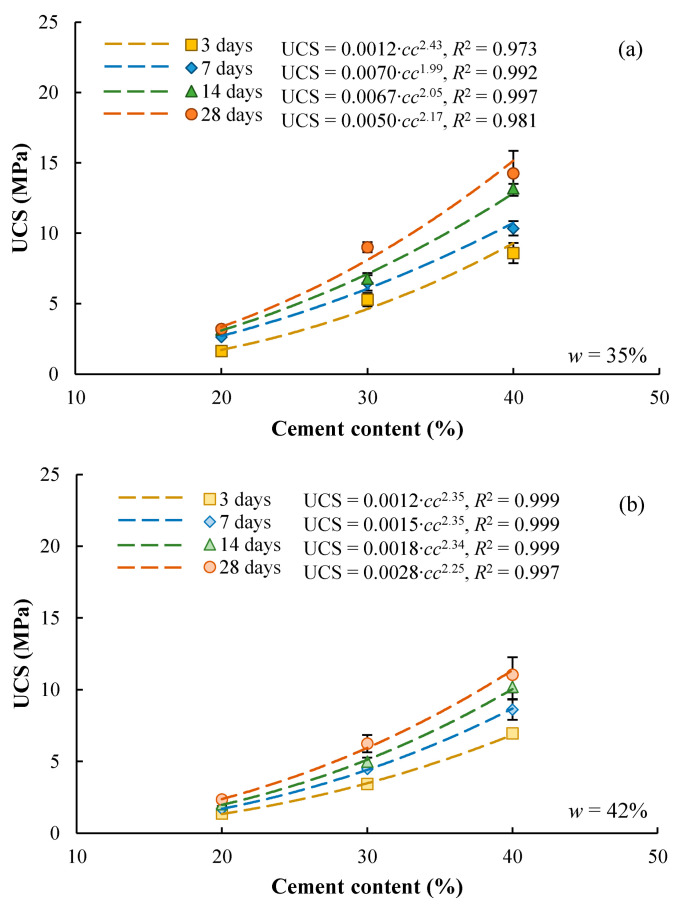
Effect of cement content on UCS of cement-stabilized Qiantang River silty clay, (**a**) mixing water content of 35% and (**b**) mixing water content of 42%.

The amount of cement used for stabilizing the soft clay is one of the most important factors. Figure 3 presents the test data of UCS-*cc* relationships for *w* = 35% (Figure 3a) and for *w* = 42% (Figure 3b). It is clear that the UCS increases with increasing cement content for a given mixing-water content and a given curing time. The following power functions were used to characterize the UCS-*cc* relationships:

(1) $$\mathrm{UCS}=A_{1}\cdot cc^{B_{1}}$$

where *A*_1_ and *B*_1_ are fitting parameters. This equation assumes that UCS is zero if no cement is added, which is reasonable for specimens reconstituted from slurries with a relatively high water content. Similar equations were also adopted by other researchers [18]. The experimental data are compared with the predicted curves in Figure 3, showing good agreements.

**Figure 4:**
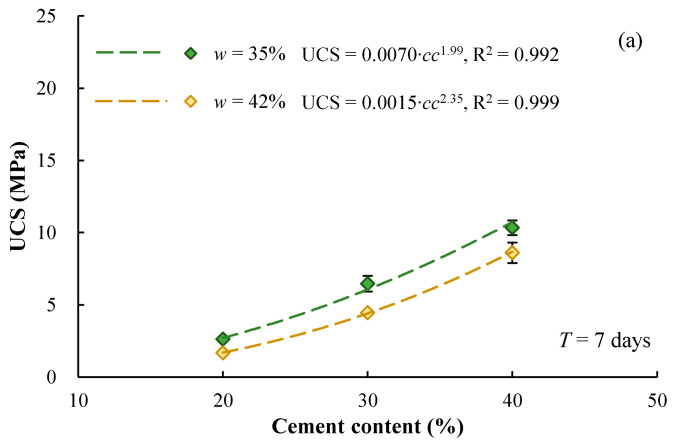

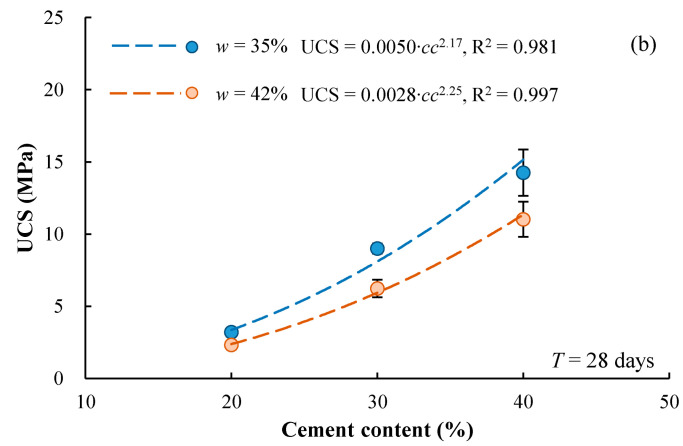
Effect of mixing-water content on UCS of cement-stabilized Qiantang River silty clay, (**a**) curing for 7 days and (**b**) curing for 28 days.

Figure 4a,b compares the UCS-*cc* relationships for specimens with different mixing-water contents, which were cured for 7 days and 28 days, respectively. It is clear that the UCS decreases with increasing mixing-water content for a given cement content. This is probably because the higher water content leads to a larger void formed between solid particles and also decreases the strength of the bonding materials. 

**Figure 5:**
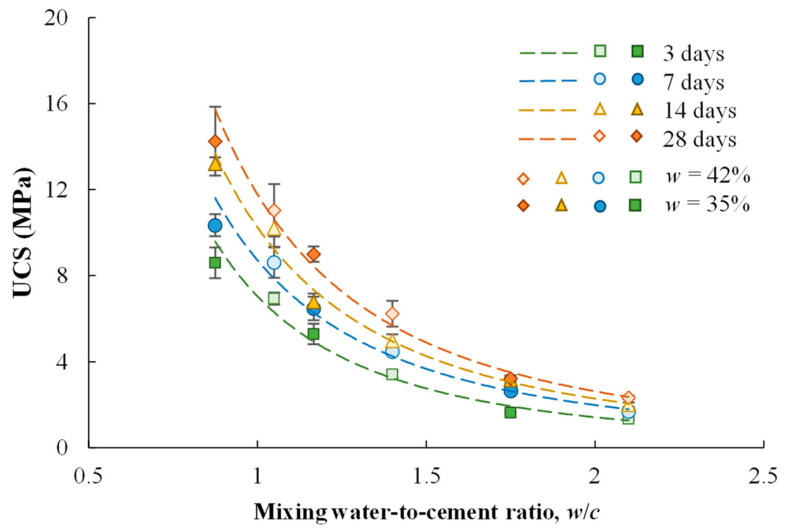
Effect of *w*/*c* on UCS of cement-stabilized Qiantang River silty clay.

**Figure 6:**
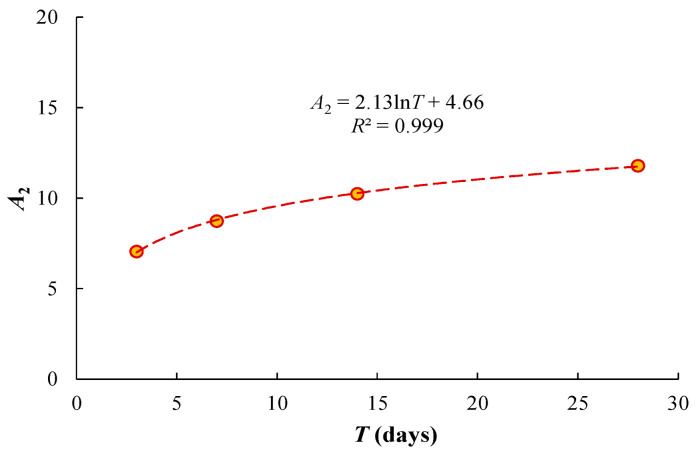
The logarithmic relationship between *A*_2_ and *T.*

Figure 5 presents the test data showing that UCS decreases with increasing *w*/*c* in a unified way for each curing duration, regardless of cement content and mixing-water content. The mixing-water-to-cement ratio is a useful index to characterize the combined effects of cement content and water content on the UCS of some cement-stabilized soils. This relationship resembles the well-known Abram’s Law for concretes. Chian et al. [14] and Lee et al. [6] adopted the following equation to characterize the UCS-*w*/*c* relationship:

(2) $$\mathrm{UCS}=A_{2}{\left(w/c\right)}^{B_{2}}$$

where *A*_2_ and *B*_2_ are fitting parameters. Unlike what was conducted in the present study, where the dry soil was mixed with water and cement, engineering practice usually adds pre-mixed cement slurry to the wet soil. For the engineering practice, the mass of water to calculate the mixing *w*/*c* includes the mass of water in the cement slurry and the mass of water in the wet soil. Table 3 summarizes the calibrated values of *A*_2_ and *B*_2_ for each curing duration. It is found that *A*_2_ increases with curing time and *B*_2_ varies slightly from −2.14 to −2.31. Figure 6 presents the effect of curing duration on the fitting parameter *A*_2_, showing that *A*_2_ increases logarithmically with *T*.

### 3.2. Effects of Curing Duration on UCS

**Figure 7:**
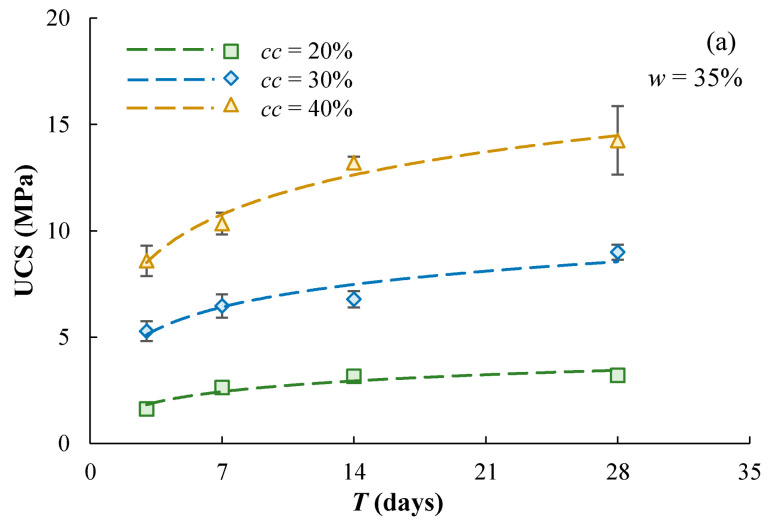

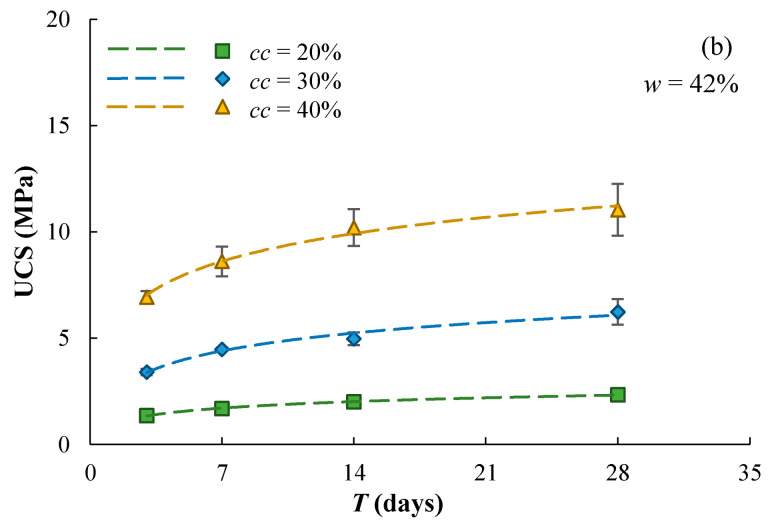
Effect of curing time on UCS of cement-stabilized Qiantang River silty clay, (**a**) mixing water content of 35% and (**b**) mixing water content of 42%.

**Figure 8:**
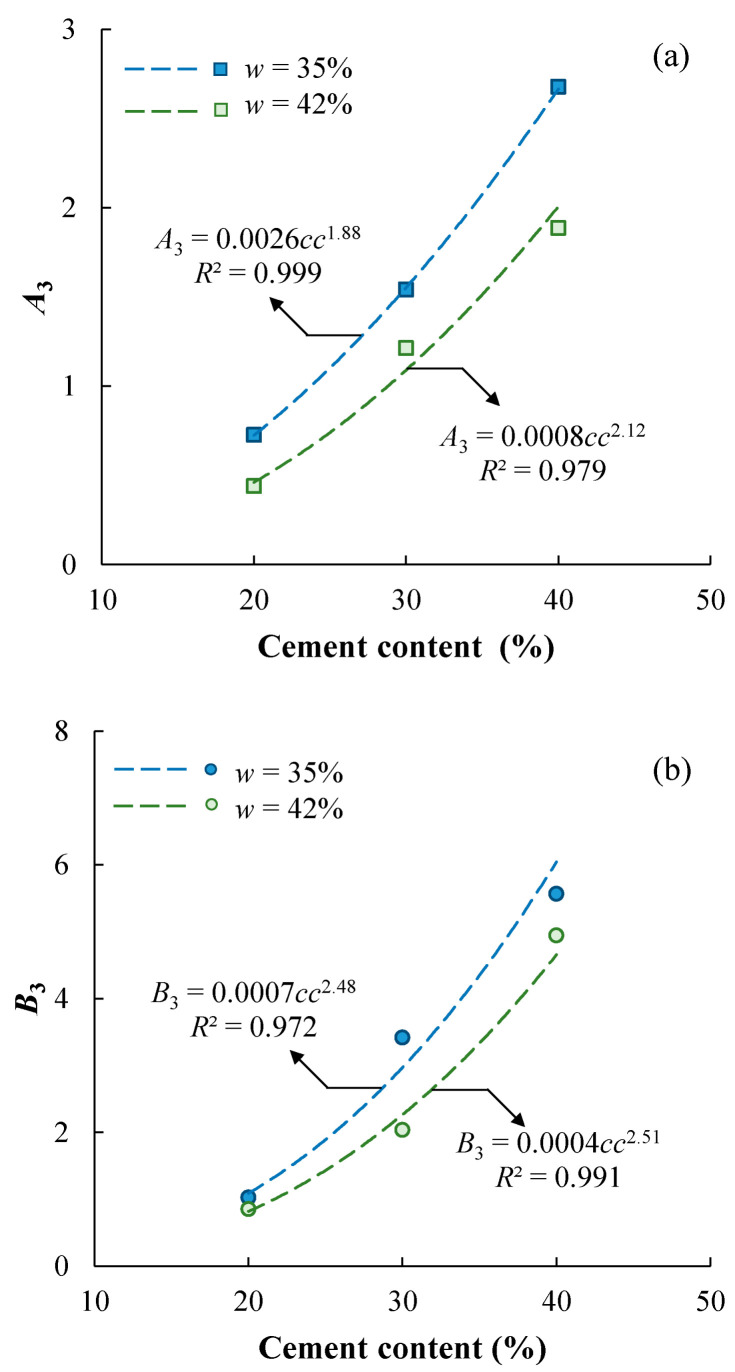
(**a**) *A*_3_ and (**b**) *B*_3_ are power functions of cement content.

**Figure 9:**
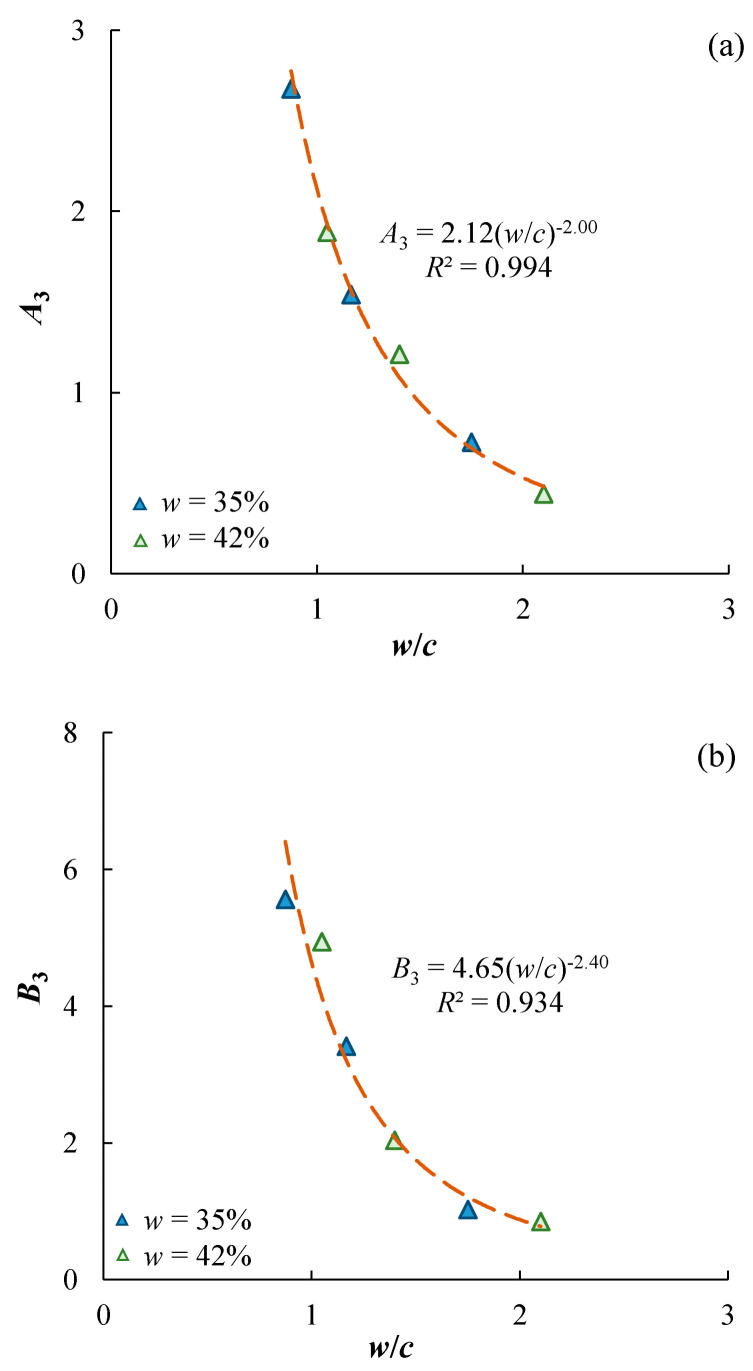
(**a**) *A*_3_ and (**b**) *B*_3_ are power functions of *w*/*c.*

The UCS of the cement-stabilized Qiantang River silty clay increases with the increasing duration of curing (Figure 7). The strength development is relatively fast in the first 7 days, mainly due to the hydration of the cement. The strength kept growing but at a relatively slow rate when specimens were cured from 7 days to 28 days, since the hydration of cement slowed down and the pozzolanic reaction started to overwhelm it. The following logarithm function can be used to capture the UCS development from *T* = 3 to 28 days:

UCS = *A*_3_ln*T* + *B*_3_ (3)

where *A*_3_ and *B*_3_ are fitting parameters; *T* is the duration of curing in days. The calibrated values of *A*_3_ and *B*_3_ are summarized in Table 4. Both *A*_3_ and *B*_3_ are functions of cement content and mixing-water content, as shown in Figure 8. They all increase with increasing *cc* and power functions can be used to characterize the trends, while they also decrease with increasing *w*. In particular, Abram’s Law applies to the two fitting parameters of the unique trends that *A*_3_ and *B*_3_ decrease with increasing mixing-water-to-cement ratio, and it can be proposed regardless of the cement content and mixing-water content (Figure 9). It should be noted that Equation (3) assumes that the value of *B*_3_ represents the UCS of the specimens cured for 1 day; however, the fitted value may not be realistic if the database does not have the test results for *T* = 1 day. In some cases, negative *B*_3_ can be obtained. For example, using Equation (3) to characterize the experimental data of Kaniraj and Havanagi [26] and Chen et al. [27] leads to unrealistic negative *B*_3_. Some researchers [14,15] would suggest *B*_3_ = 0 for simplicity and to maintain the capability of characterizing the UCS-*T* data for longer curing durations.

### 3.3. Prediction of UCS of the Cement-Stabilized Qiantang River Silty Clay

**Figure 10:**
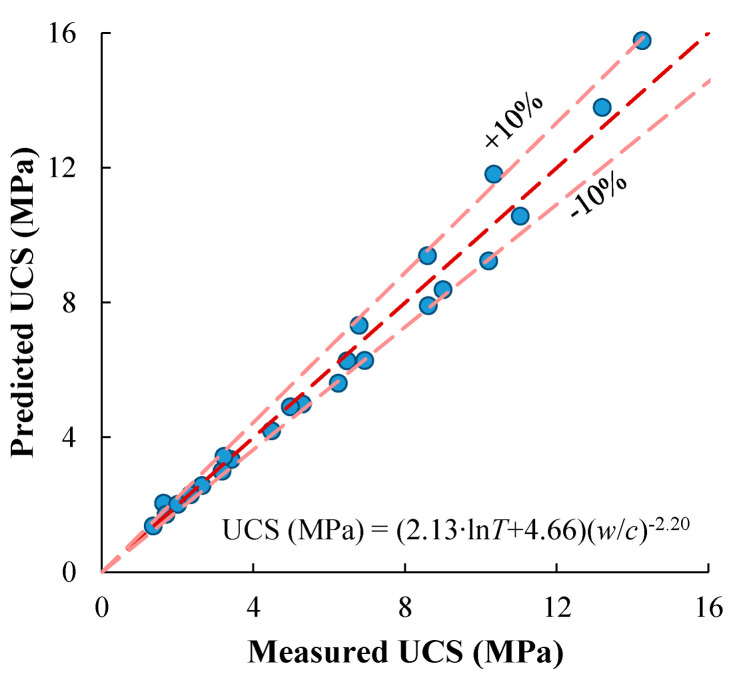
Comparing the measured and predicted UCSs.

For the cement-stabilized silty clay in this study, the mixing-water-to-cement ratio and curing duration are two major factors controlling the unconfined compressive strength. According to the previous parametric study, the following equation is proposed to characterize the effects of *w*/*c* and *T* on UCS:

(4) $$\mathrm{UCS}=\left(A_{4}\ln T+B_{4}\right){\left(w/c\right)}^{C_{4}}$$

where *A*_4_, *B*_4_, *C*_4_ are fitting parameters, with values of 2.13, 4.66, and −2.20, respectively. The predicted UCS are compared with the measured values in Figure 10, showing fairly good agreement. 

**Figure 11:**
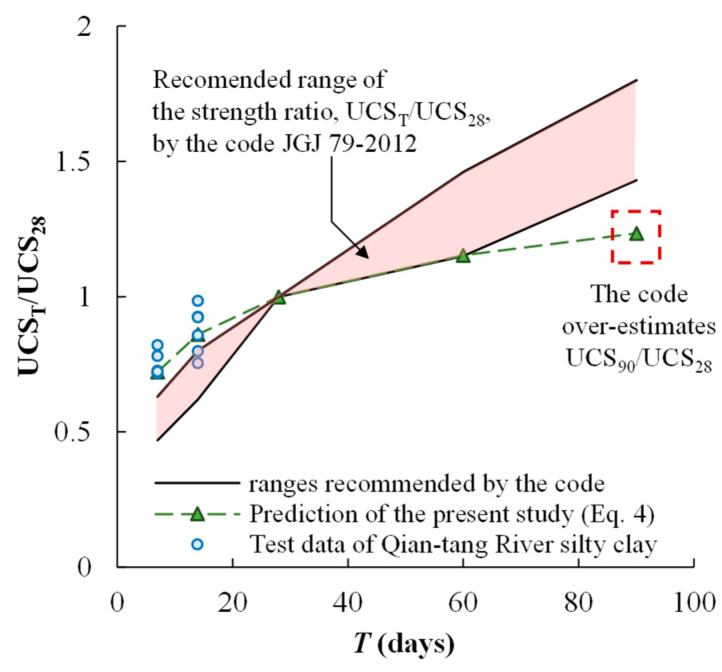
Comparing the code-recommended UCS_T_/UCS_28_-*T* relationship to the tested data and the prediction curve of the cemented Qiantang River silty clay.

From a practical point of view, proper characterization of the effect of curing duration on UCS is important for the prediction of long-term strength and the evaluation of the safety capacity of the designed strength. A technical code used in China, Technical Code for Ground Treatment of Buildings (JGJ-79-2012 [28]), suggested that the long-term UCS can be predicted using the UCS cured for 28 days (UCS_28_) if it is not feasible time-wise to measure the UCS cured for 90 days. The code also recommended the ranges of a factor that is multiplied to UCS_28_ to predict UCS_7_, UCS_14_, UCS_60_, and UCS_90_ (i.e., UCS cured at 7, 14, 60, and 90 days, respectively). Figure 11 compares the range recommended by the code with the prediction curve derived from the present study. It is shown that the recommendation of the code can significantly overestimate the long-term strength of Qian-tang River silty clay (UCS_60_ and UCS_90_) using UCS_28_, leading to an unsafe design. The results shown in Figure 11 indicate a need for modification of the technical code.

## 4. Discussion

Similar to the experimental data reported in the literature, the UCS of cement-stabilized Qiantang River silty clay increases with the increasing cement content, decreasing mixing-water content, and increasing curing duration under otherwise similar conditions. In particular, the UCS of cement-stabilized soil in the present study decreases with an increasing mixing-water-to-cement ratio for a given curing time, regardless of cement content and water content. This agrees with the cement-stabilized Bangkok clay reported by Horpibulsuk et al. [5]. However, Lee et al. [6] found that the UCS of cement-stabilized Singapore marine clay mainly decreases with increasing *w*/*c*, while the UCS is also affected by the ratio between the mass of soil to the mass of cement (i.e., the inverse of *cc*). Chian et al. [14] reported experimental findings similar to those of Lee et al. [6]. Noting that the soils tested using these investigation measures are from different regions of the world, and the abovementioned difference could be due to the different mineralogy. In addition, those investigations also used different types of cement, introducing additional uncertainties. A series of unconfined compression tests were conducted using cement-stabilized kaolin clay. The basic properties of kaolin clay are given in Table 1. The testing procedures followed those for cement-stabilized Qiantang River silty clay. 

**Figure 12:**
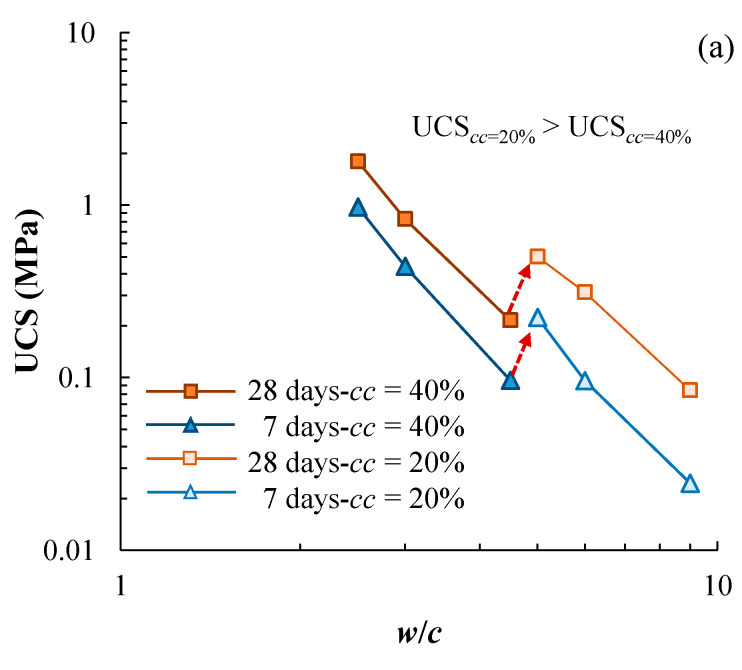

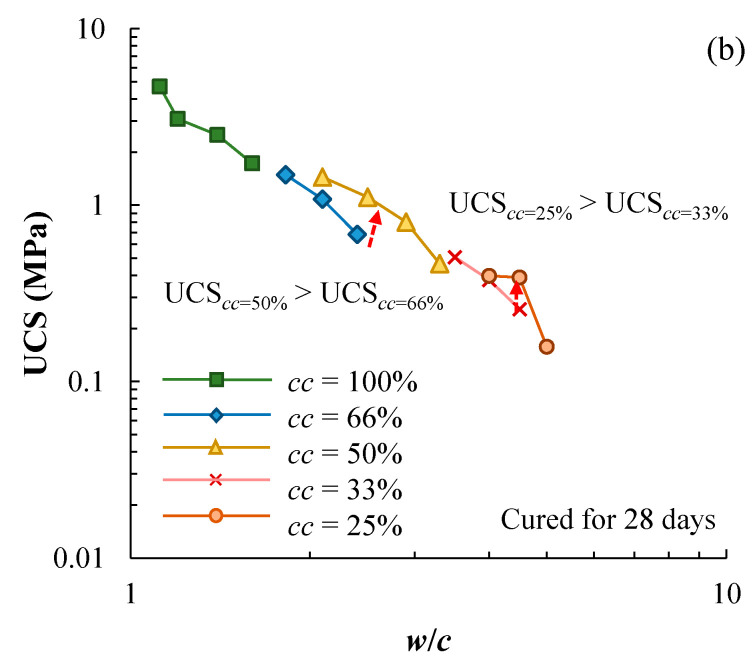
Effect of cement content on the UCS-*w*/*c* relationships for (**a**) cement-stabilized kaolin clay in the present study and (**b**) cement-stabilized Singapore marine clay (data from Lee et al. [6]).

The UCS-*w*/*c* data of cement-stabilized kaolin clay is presented in Figure 12a. For a given curing duration, two different trend lines can be proposed for *cc* = 40% and 20%. In particular, the data for *cc* = 20% are above those for *cc* = 40%. Clearly, cement content has a certain level of impact on the UCS-*w*/*c* relationship, which is inconsistent with the observation for the cement-stabilized Qiantang River silty clay. Noting that the same cement was used and the same testing procedures were followed for the two types of cement-stabilized clays, the difference between the two base soils is the cause of the inconsistent observation. Figure 12b presents the literature data of cement-stabilized Singapore marine clay, showing that the UCS-*w*/*c* is affected by cement content to some extent, and the UCS-*w*/*c* relationship moves upwards with decreasing cement content. Lee et al. [6] claimed that the flocculation of the soil particles due to the addition of cement leads to the cement-content-dependent UCS-*w*/*c*.

**Figure 13:**
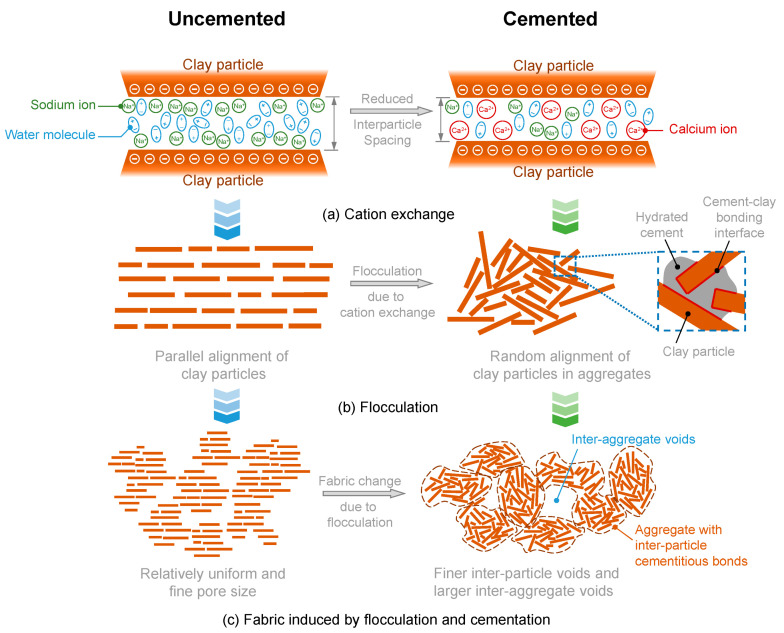
Schematics of the formation of the fabric of the cemented clay.

The mechanism is schematically explained in Figure 13. The hydration of cement produces calcium hydroxide, and the calcium ions replace the monovalent cations (e.g., sodium ions) on the surface of clay particles to reduce the inter-particle distance (Figure 13a). The cation exchange causes flocculation of the clay particle, making the regularly oriented particles become randomly oriented and form aggregates (Figure 13b). Inside the aggregates, particles are bonded using the hydrated cementitious materials. The flocculation eventually leads to a different fabric compared to the uncemented clay (Figure 13c). Kamruzzaman et al. [29] measured the pore size distribution of the cement-stabilized clay and found that increasing cement content (with a fixed mixing-water content) leads to larger pore size and a more even distribution of pore size. This probably suggests that if compared at the same *w*/*c*, the higher cement content may lead to an even larger pore size due to stronger flocculation and higher water content. The inter-aggregate connection is the major component that sustains loading. Larger pore size indicates fewer inter-aggregate connections, and thus, a relatively less-stable structure is anticipated at the same *w*/*c* but higher cement content. For soils with more clayey minerals and higher plasticity, the flocculation due to cation exchange may have a higher impact. This may explain why the UCS-*w*/*c* of Qian-tang silty clay is not affected by the cement content, while the UCS-*w*/*c* of Kaolin clay is affected to some extent. 

**Figure 14:**
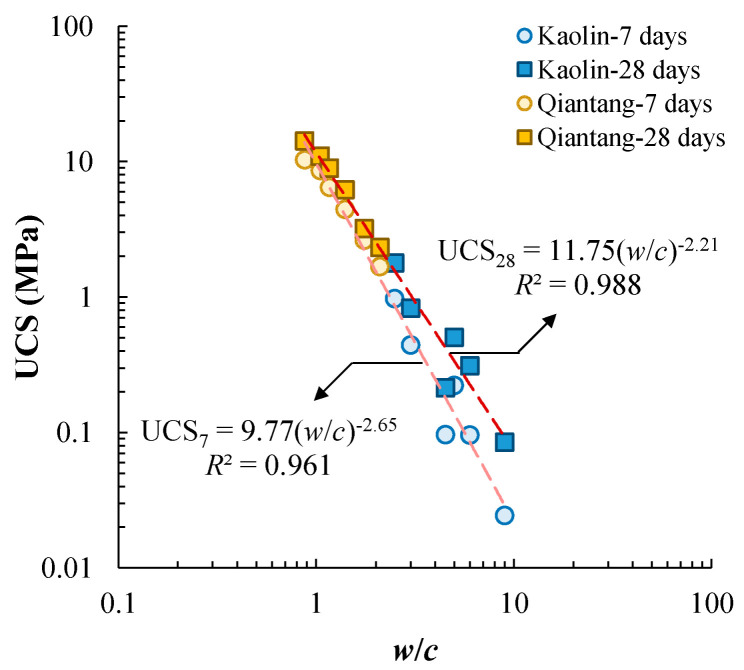
Unique UCS-*w*/*c* relationship of cemented Qiantang River silty clay and cemented Kaolin clay.

**Figure 15:**
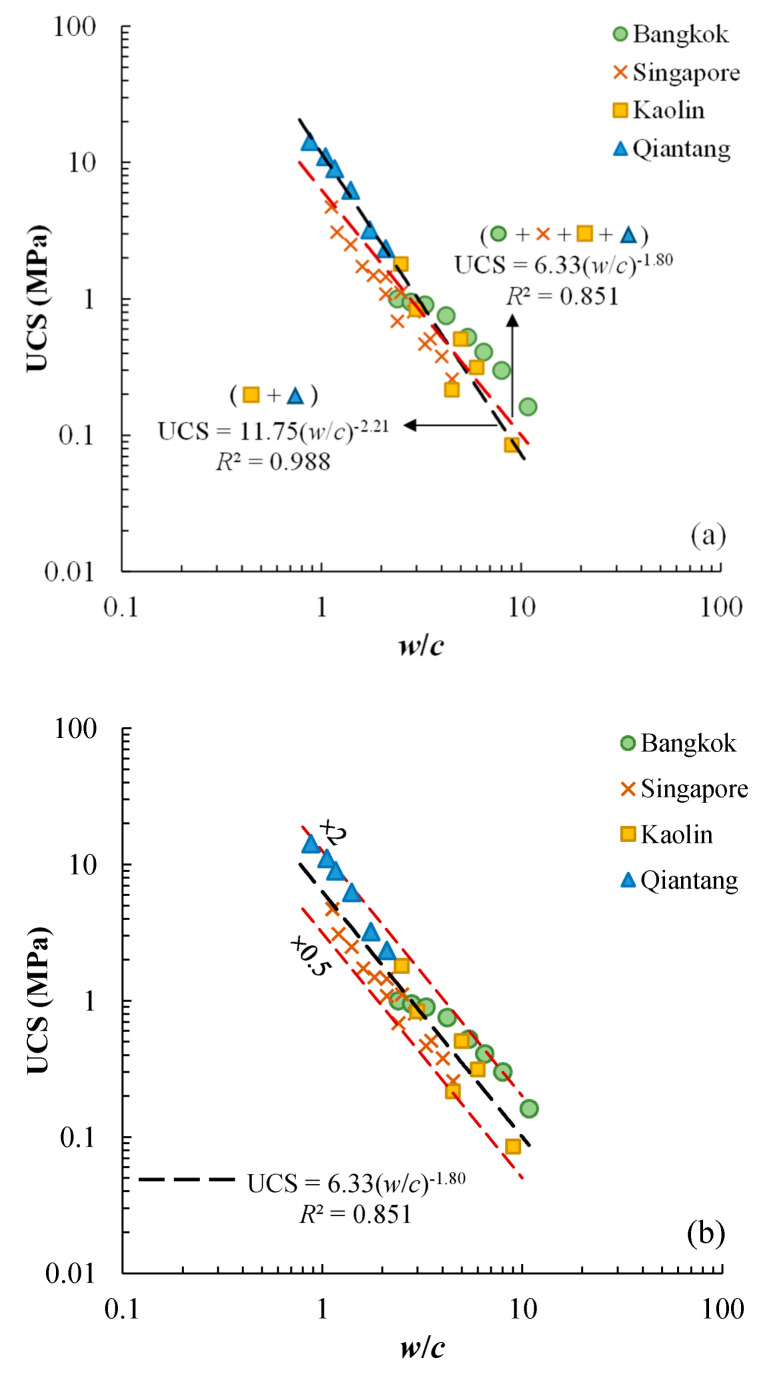
Unified UCS-*w*/*c* relationship of cemented clays of the present study and the literature (*T* = 28 d), (**a**) comparing the trend of data from the present study and the trend of four cement-stabilized clays; (**b**) all data fall into the vicinity of proposed trend of the four cement-stabilized clays

It should be noted that the effect of cement content on the UCS-*w*/*c* relationship is relatively minor and can be ignored, and a similar suggestion has been made by Chian and Bi [30] from a practical perspective. Figure 14 compares the UCS-*w*/*c* relationships of the cement-stabilized Qiantang River silty clay and the cement-stabilized kaolin clay, cured for 7 days and 28 days. For each curing duration, a more-or-less unified UCS-*w*/*c* relationship can be proposed if the effect of cement on the UCS-*w*/*c* relationship of cement-stabilized kaolin clay is ignored. Figure 15 compares the UCS-*w*/*c* relationships for the test data of the present study and the literature data. All the data points fall into a relatively narrow band, showing a more-or-less unified relationship. The trend lines for the stabilized Qiantang River silty clay and kaolin are also compared with all four types of stabilized clays. Some other investigations show that different UCS-*w*/*c* relationships can be obtained for different types of clays. Chian and Bi [30] investigated the UCS of a series of cement-stabilized sand-clay mixtures (i.e., sandy clays). They found that UCS decreases with increasing *w*/*c* monotonically for a given sand type and sand content, but the UCS-*w*/*c* correlation of different sand types and sand contents may not necessarily be unique. Finally, they proposed a unified method to characterize the UCS of all types of cemented sandy clays using a free-water-to-cement ratio, (*w*/*w*_L_)/*c*. Similarly, Zhang et al. [9] also proposed a unified prediction method of UCS of three types of sandy clays incorporating water content, cement content, and liquid limit. The unified UCS-*w*/*c* relationship suggested by the present study is only applicable to clay without a substantial amount of sand. 

Although Abram’s law can be used to characterize the UCS of cement-stabilized clay, it should be noted that some cemented soils do not follow Abram’s law. Wei et al. [31] reported that the void ratio of the cement-stabilized soil can affect the UCS-*w*/*c* relationships. Two possible reasons can be identified, the different base soil and the different specimen preparation methods. Wei et al. [31] used clayey sand, which is very different from the clays used in this study. In addition, they prepared specimens by compacting wet soil–cement mixture to different void ratios. For the soils tested in the present study, the specimens were solidified from a cement-soil slurry, the void ratio is controlled by the water added during mixing. These differences lead to different micro-structures of the soils. In addition, the proposed equation is only valid for cement-stabilized clay with a mixing-water content close to and higher than the liquid limit of the clay.

## 5. Conclusions

This study investigated the unconfined compressive strength of a cement-stabilized Qian-tang River silty clay, by considering different cement contents, mixing-water contents, water-to-cement ratios, and curing durations. The major findings are given as follows:

1. The UCS of the cement-stabilized Qian-tang River silty clay increases with increasing cement content and decreases with increasing mixing-water content, under otherwise similar conditions.
2. For a given curing duration, the UCS of the cement-stabilized Qian-tang River silty clay decreases with increasing water-to-cement ratio uniquely, regardless of mixing-water content and cement content. For each of the curing times, UCS increases by around five times when the water-to-cement ratio reduces from 2.1 to 0.875.
3. There appears to be a unified UCS-*w*/*c* correlation for a given curing duration, through an analysis of several cement-stabilized clays. All data points fall into a band confined by 2 times and 0.5 times the average trend.
4. A prediction method of UCS is proposed by considering the water-to-cement ratio and curing time for the Qian-tang River silty clay.
5. The experimental data of the present study suggested that using the method recommended by the technical standard in China to predict the UCS may overestimate the strength cured for 90 days, which is the design value for an engineering project.

## Figures and Tables

**Table 1:** Basic physical properties of soil.

Type of Soil	Liquid Limit *w*_L_ (%)	Plastic Limit *w*_P_ (%)	Plasticity Index *I*_p_ (%)	Soil Classification
Qiantang River silty clay	33.1	16.2	17.0	CL
Kaolin clay	60.5	33.5	27.0	CH

**Table 2:** Test program of Qiantang River silt clay.

Mixing-Water Content *w* (%)	Cement Content *cc* (%)	Mixing-Water-to-Cement Ratio *w*/*c*	Curing Duration *T* (days)
35	20	1.75	3, 7, 14, 28
35	30	1.17	3, 7, 14, 28
35	40	0.88	3, 7, 14, 28
42	20	2.10	3, 7, 14, 28
42	30	1.40	3, 7, 14, 28
42	40	1.05	3, 7, 14, 28

**Table 3:** Calibrated fitting functions of UCS-*w*/*c* relationships of cemented Qiantang River silty clay.

Curing Duration, *T* (days)	Fitting Equation	*A*_2_	*B*_2_	*R* ^2^
3	UCS = 7.05∙(*w/c*)^−2.31^	7.05	−2.31	0.990
7	UCS = 8.73∙(*w/c*)^−2.14^	8.73	−2.14	0.992
14	UCS = 10.25∙(*w/c*)^−2.17^	10.25	−2.17	0.974
28	UCS = 11.79∙(*w/c*)^−2.17^	11.79	−2.17	0.998

**Table 4:** Fitting functions of USC of cemented Qiantang River silty clay and *T.*

Mixing-Water Content *w* (%)	Cement Content *cc* (%)	Fitting Equation	*A*_3_	*B*_3_	*R* ^2^
35	20	UCS = 0.73∙ln*T* + 1.02	0.73	1.02	0.889
35	30	UCS = 1.54∙ln*T* + 3.42	1.54	3.42	0.900
35	40	UCS = 2.68∙ln*T* + 5.56	2.68	5.56	0.972
42	20	UCS = 0.44∙ln*T* + 0.85	0.44	0.85	0.998
42	30	UCS = 1.21∙ln*T* + 2.04	1.21	2.04	0.976
42	40	UCS = 1.88∙ln*T* + 4.94	1.88	4.94	0.988

## Data Availability

Data are contained within the article.
